# Patient-reported outcomes in sarcopenia: An ICFSR task force report

**DOI:** 10.1016/j.tjfa.2024.100010

**Published:** 2025-01-01

**Authors:** Charlotte Beaudart, David Cella, Roger A. Fielding, Yves Rolland, Bruno Vellas, Marco Canevelli, ICSFR Task Force

**Affiliations:** a Clinical Pharmacology and Toxicology Research Unit (URCP), Namur Research Institute for Life Sciences (NARILIS), Department of Biomedical Sciences, Faculty of Medicine, University of Namur, Namur, Belgium; b Department of Medical Social Sciences, Feinberg School of Medicine, Northwestern University, Chicago, IL, USA; c Nutrition, Exercise Physiology and Sarcopenia Laboratory, Jean Mayer USDA Human Nutrition Research Center on Aging at Tufts, Boston University, Boston, MA, USA; d IHU HealthAge CHU Toulouse, Toulouse, France; e Department of Human Neuroscience, Sapienza University, Rome, Italy; f National Center for Disease Prevention and Health Promotion, Italian National Institute of Health, Rome, Italy; g Aging Research Center, Department of Neurobiology, Care Sciences, and Society, Karolinska Institute and Stockholm University, Stockholm, Sweden

**Keywords:** Sarcopenia, PROMs, Quality of life, Clinical trials, Patient-centered care

## Abstract

The International Conference on Frailty and Sarcopenia Research (ICFSR) Task Force convened in March 2024 to address patient-reported outcomes measures (PROMs) in the field of sarcopenia. PROMs are crucial to enhance healthcare services at both individual and societal levels. PROMs complement objective outcome measures by capturing insights that patients are best suited to judge. In recent years, there has been an increase in the recognition of PROMs’ importance within clinical trials by pharmaceutical industries and regulatory agencies. Consequently, it has become imperative to develop valid and reliable tools tailored to capture various aspects of patient's experience and health status. This report aims to present the state-of-the-art available and validated PROMs for sarcopenia that can be used within clinical settings by various stakeholders, and to highlight several research gaps and barriers that need to be addressed to expedite and improve the use of these outcome measures within the context of clinical trials.

## 1 Introduction

The International Conference on Frailty and Sarcopenia Research (ICFSR) Task Force convened in March 2024 to discuss issues related to translational research on mitochondrial aging, drug development in sarcopenia and frailty, and the utilization of patient-reported outcomes in clinical trial of sarcopenia and frailty. Task Force participants presented state of the art updates that were followed by a robust discussion of key issues that will be required for regulatory approval of new medicines to prevent and treat sarcopenia and frailty.

Recognizing the need to accelerate the development of treatments for sarcopenia, improve health outcome assessments, and to integrate the shift of health care models to a more patient-centric approach, the ICFSR Task Force gathers relevant experts from academia and industry, across multiple professional backgrounds, from 16 countries in North America, South America, Europe, Asia, and Australia/Oceania to address the topic of patient-reported outcomes measures in the field of sarcopenia. The present article reports on the main outputs from this ICFSR Task Force held in Albuquerque in 2024.

## 2 Patient-reported outcome measure: definition and importance

Over the past two decades, there has been a notable shift in health systems towards a more patient-centered model of care [1]. The Institute of Medicine has defined patient-centered care as “care that is respectful of and responsive to individual patient preferences, needs, and values” [2]. This transition has been driven by various stakeholders including clinicians, pharmaceutical industries, and regulatory agencies, all of whom have come to recognize the importance of integrating patient-reported outcomes measures (PROMs) alongside traditional biomarkers of health improvement [[3], [4], [5]]. This recognition has underscored the significance of considering not only clinical indicators but also the subjective experiences and perspectives of patients [2].

This paradigm shift has necessitated the development of identifiable, valid, and reliable tools, tailored to capture different facets of patient experience and health status. In the whole framework of clinical outcome assessment (COA), different tools have been described. The most reported ones are those that target the patients directly, i.e. PROMs and patient-reported experience measures (PREMs). PROMs aim to report on diseases and symptoms, treatment side effects (i.e. pain, fatigue, or anxiety), functional outcomes (i.e. physical, sexual, social, role, emotional, or cognitive functioning), or multidimensional constructs like health-related quality of life (HRQoL) or health utility. PREMs evaluate the overall experiences of patients within the healthcare system, including interactions with healthcare providers and the accessibility of services. Besides these patient-administered tools, Clinician-Reported Outcomes measures (ClinROs) provide insights into health outcomes from the perspective of clinicians or healthcare professionals, drawing on their clinical observations and assessments, Observer-Reported Outcomes measures (ObsROs) are akin to ClinROs but involve health outcomes observed and reported by external observers, such as caregivers or family members and, lastly, Performance Outcome Measures (PerfOs), assess the effectiveness of specific interventions or treatments by measuring performance or outcomes objectively (https://www.fda.gov/about-fda/division-patient-centered-development/clinical-outcome-assessments-coas-medical-device-decision-making). Collectively, these instruments contribute to a comprehensive understanding of patient well-being, treatment effectiveness, healthcare experiences, and thereby facilitating informed decision-making as well as improving the overall quality of care.

In the field of PROMs, two main approaches, namely generic and disease-specific instruments, can be used. Generic PROMs are designed to be applicable to diverse populations of any age and with various health conditions. These instruments are widely utilized in both observational studies and clinical trials because they allow for comparisons across different populations, such as comparing the impact of a disease on HRQoL across various stages of the disease or comparing HRQoL between different diseases. For instance, generic questionnaires like the Short-Form 36 questionnaire (SF-36) [3], the EuroQoL 5-dimension (EQ-5D) questionnaire [4], and the EQ visual analog scale (EQ-VAS) questionnaire are commonly used in research. On the other hand, disease-specific PROMs are tailored to measure PRO in individuals with a particular health condition. These instruments address aspects of life affected by the specific disease. This specificity can sometimes provide a more focused assessment of the disease's impact when evaluating treatment effectiveness or disease progression. Many disease-specific PRO have been developed in the past few years to assess HRQoL [5].

Both generic and disease specific instruments are important and can be used in combination to offer a broader investigation of the impact of one health condition on PRO. These diverse instruments play a crucial role in capturing the complexities of PRO and providing valuable insights into the impact of health conditions on individuals' lives.

## 3 HRQoL in sarcopenia

### 3.1 What does the literature say?

**Fig. 1:**
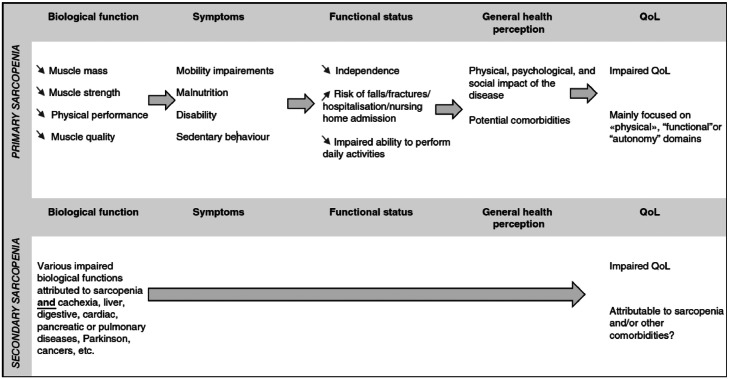
Conceptual model of QoL in sarcopenia proposed by Beaudart et al. [16]. (reused with permission).

Sarcopenia is characterized by an age-associated loss of skeletal muscle mass and function, is now recognized as a disease entity and figures in The International Statistical Classification of Diseases and Related Health Problems - Clinical Modification Code (ICD-10-CM, code M62.84) [6,7,8]. This multifactorial disease is associated with an increased likelihood of adverse outcomes. It is nowadays well recognized that the risk of functional decline, falls, fractures, hospitalizations, and even death increase in individuals with sarcopenia [[9], [10], [11], [12]]. While these investigations have mainly focused on so-called “hard clinical outcomes”, there has also been a growing interest in the lived experience of people with sarcopenia. One of the most explored types of PROM is HRQoL. The World Health Organization (WHO) has proposed a broad definition of QoL, conceptualized as “*the individual's perception of their position in life in the context of the culture and value systems in which they live and in relation to their goals*”. Many conceptual models for HRQoL have been elaborated, including the Wilson & Cleary model, the Ferrans et al. [13]. model (a revision of the Wilson & Cleary model), and the WHO models. In 2021, Beaudart et al. [14]. proposed a conceptual model of QoL in sarcopenia based on the model of Ferrans et al. [15]. (Fig. 1. Reused with permission). The biological/physiological functions impacted by sarcopenia are responsible for mobility impairments, disability, and sedentary behaviour symptoms that impact the functional status and contribute to a lower QoL in sarcopenic individuals. HRQoL measures have been shown to be significant predictors of hard clinical outcomes, such as hospitalization or mortality, reinforcing the importance of their assessment.

Indeed, there is a consensus on the negative HRQoL impact of age-related sarcopenia, as highlighted by a meta-analysis including 43 observational studies reporting an assessment of HRQoL in 4108 sarcopenic individuals in comparison with 26,214 healthy individuals [17]. A pooled standardized mean difference (SMD) of 0.76 (95 % CI 0.95, 0.57) was found, indicating significantly reduced QoL in sarcopenic individuals. While authors did not report the different magnitude of effect size across various age groups, sarcopenia diagnosis definitions, or regions/countries/continents, a larger SMD was observed when analyses were restricted to studies using disease-specific instruments (SMD of 1.09) as compared to studies using generic ones (SMD of 0.49, interaction p-value <0.01). Among the 43 studies, 20 used the specific Sarcopenia and Quality of Life questionnaire (i.e. SarQoL) whereas 23 used a generic instrument (i.e., SF-36 *n* = 11, EQ 5D *n* = 8, others *n* = 5).

### 3.2 Sarcopenia-specific PROMs

Currently, three different PROMs specific to sarcopenia co-exist in the scientific literature (Table 1). The first one, developed by Evans et al. in 2011 [15], namely the Age-Related Muscle Loss Questionnaire (ARMLQ) can be used in both clinical practice and clinical trial settings to the patient's perspective of the functional impacts of reduced muscle strength in sarcopenia. This instrument has been developed according to the Consensus-based Standards for the selection of health Measurement Instruments (COSMIN) recommendations [16] and is composed of 14 questions. However, the instrument has not been tested yet for its psychometric properties (i.e. validity, reliability, and responsiveness).

**Table 1:** Review of existing specific PROMs for sarcopenia.

Name of the PROM	Investigated construct	Characteristic of the PROM	Development of the PROM	COSMIN Clinimetric properties		
				Validity	Reliability	Responsiveness
**ARMQoL** [15]	Functional impacts of reduced muscle strength	14 items, Likert scale.	Literature review, input from experts, patients’ interviews	**Content validity:** confirmed by open-ended, concept elicitation interviews with 12 patients with sarcopenia. Not confirmed by healthcare professionals’ interviews [15]. **Construct validity:** NR	**Internal consistency:** NR **Test-retest reliability:** NR **SEM:** NR	**Responsiveness to change:** NR
**SarQoL**[18], [19], [20], [21]	Quality of life	55 questions, 22 items, 10–15 min of administration. 7 domains of HRQoL. A short form has been developed including 14 items.	Literature review, experts semi-structured questionnaire, patients interviews	**Content validity:** confirmed by a recent a posteriori content validity analysis including 17 patients with sarcopenia and 11 healthcare professionals [22]. **Construct validity:** divergent and convergent validity confirmed in 19 validation studies [19].	**Internal consistency:** confirmed with Cronbach alpha >0.8 consensually found in 19 different validation studies [19]. **Test-retest reliability: confirmed with** ICC >0.9 found in 18 out of 19 validation studies [19]. **SEM:** 2.65 points obtained from the pooling of 9 cohort studies (i.e. 278 individuals with sarcopenia) [23].	**Responsiveness to change:** confirmed in two observational prospective studies. Superiority to generic instruments was also reported. Responsiveness to change following an intervention is still lacking [24].
**PROMIS physical function item bank** [25,26]	Wide range of physical function abilities and limitations	163 item bank with multiple 4–20 item-long short form options as well as computer adaptive testing	Literature review, expert input, patient interviews	**Content validity:** confirmed in patient interviews, surveys, and cognitive debriefing. **Construct validity:** divergent and convergent validity confirmed in multiple validation studies; sufficient unidimensionality and item response theory model fit	Multiple studies confirmed high internal consistency of multiple short forms. CAT reliability exceeds 0.95. Test-retest reliability above 0.70 in multiple studies	Responsive to change associated with drug and behavioral interventions across several conditions. Performance in sarcopenia to be determined.

SEM: standard error of measurement; ICC: intra-class coefficient correlation; NR: not reported.

The second PROM specific to sarcopenia available in the scientific literature is the SarQoL questionnaire [18,27]. This self-administered instrument aiming to measure the construct of HRQoL specific to sarcopenia, developed in 2015, consists of 55 items arranged into 22 questions and has been translated into 35 languages (http://www.sarqol.org). The questionnaire is scored, through a scoring algorithm, on 100 points, with higher scores reflecting a better QoL. Items are organized into seven domains of HRQoL reflecting the overall quality of life of the individuals: domain 1 “Physical and Mental Health”; domain 2 “Locomotion”; domain 3 “Body Composition”; domain 4 “Functionality”; domain 5 “Activities of daily living”; domain 6 “Leisure activities”; and domain 7 “Fears”. SarQoL is freely available for clinical and research purposes from the website www.sarqol.org. Up to now, SarQoL is the only validated specific HRQoL questionnaire for sarcopenia. Since its development, 19 validation studies performed on SarQoL to detect differences in HRQoL between individuals with and without sarcopenia, as well as its reliability, and its validity [19]. The psychometric properties of this questionnaire were analyzed according to the taxonomy of the COSMIN [28]. Two further observational studies have also indicated its responsiveness to change. Importantly, these studies mentioned a higher responsiveness of SarQoL relative to common generic tools such as the SF-36 or the EQ 5D.

A third PROM is currently being validated for use in sarcopenia and provides an interesting option. The Patient-Reported Outcomes Measurement Information System (PROMIS®) is a list of self-reported measures covering multiple domains within physical, mental, and social health [25]. They have been developed as item banks, allowing for computerized adaptive testing, as well as the extraction of short form questionnaires. The flexibility of item response theory-developed measurement systems like PROMIS allow for increased relevance and responsiveness to specific health conditions. This is accomplished by identifying, from a large bank of calibrated questions regarding a specific symptom or functional domain, items that are specifically relevant to a given diagnosis, such as age-related sarcopenia. Currently, a project funded by the 10.13039/100009210Food and Drug Administration (1U01FD006887–01) is underway to certify the PROMIS measure of physical function as a clinical outcome assessment and to investigate the specific context of use in which it could serve as a primary outcome in registration trials.

### 3.3 Use of PROMs in research and clinical settings

The decision to use a PROM should be guided by two primary considerations. Firstly, the construct being assessed, as PROMs can target a wide range of constructs. For instance, if the focus is on quality of life, it is imperative to select a PROM specifically designed for this purpose. Secondly, it is essential to ensure that the PROM has undergone proper development and validation. Methodological considerations have gained increasing significance in recent years, with COSMIN offering a framework for developing and assessing the psychometric qualities of PROMs. The COSMIN taxonomy [28,29] delineates three key psychometric properties: i) validity (encompassing content validity, construct validity and criterion validity), ii) reliability (including internal consistency, test-retest reliability, and measurement error), and iii) responsiveness to change. It is therefore essential to verify that the PROM one wishes to use has been reported with adequate content validity, including the involvement of patients in the item generation process to ensure relevance, comprehensiveness, and comprehensibility of the included items [16]. Additionally, adequate construct validity should be ensured, confirming that the included items effectively measure the intended concept of the PROM. Internal consistency reflects the extent to which items within an instrument measure various aspects of the same characteristic or construct. It is a form of reliability, often reported alongside test-retest reliability. In this context, it is also important to ensure that the standard error of measurement and the smallest detectable change values have been reported for the intended PROM. The smallest detectable change indicates the minimum amount of change in the PROM score that needs to be observed before we can be sure that the change is real and not, potentially, a result of measurement error. Recently, there have been calls to reconceptualize the validation of PROMs as an ongoing, iterative process of evidence accumulation [5]. Finally, in the realm of clinical trials, responsiveness to change is crucial as it enables researchers to gauge a PROM's capacity to detect clinically meaningful changes over time. A PROM with high responsiveness to change can capture even subtle improvements or deteriorations in patient outcomes, offering valuable insights into the efficacy of the intervention under evaluation. Although this property is less frequently reported, as its assessment necessitates longitudinal cohort studies, it is imperative to ensure that the PROM has been evaluated for adequate responsiveness to change before its application, both in observational and interventional research settings. It is nevertheless important to consider that some PROMs may not be originally developed for use in clinical practice or to inform policy decisions and may have issues with responsiveness to change or potential floor and ceiling effects that limit their potential in these settings.

## 4 Use of PROM in interventional studies for sarcopenia

Results from the meta-analysis of Beaudart et al. [17]. provide an understanding of the impact of sarcopenia on HRQoL which is important for healthcare providers and regulators as this may guide the development of care strategies for sarcopenic patients. Nevertheless, the evidence from this work is limited to observational studies. While the descriptive epidemiology of sarcopenia is now well-explored, interventional research in sarcopenia remains underdeveloped. The current approach to manage sarcopenia involves a multifaceted strategy to mitigate its impact on individuals’ health and well-being. These strategies incorporate a blend of nutritional (i.e. protein supplementation), exercise (i.e. strength and resistance training), and pharmacological strategies [[30], [31], [32], [33], [34]]. However, while sarcopenia has been recognized as an independent disease by an ICD-10-CM code [6] and is recognized by the scientific community and by clinicians as leading to adverse impact on human health and life, therefore fulfilling the FDA definition’ criteria of an indication, there is still no medication approved for this indication on the market [35]. Pharmacological clinical trials conducted in patients with sarcopenia have only progressed as far as phase II, according to a recent review [36]. Several reasons may explain the regulatory issues/limitations in this field, as discussed in a recent ICFSR report [37] One of the major limitations of clinical trials in the field of sarcopenia is the lack of a consensual diagnosis definition. Nevertheless, progress is expected, as with the forthcoming global definition by the newly formed Global Leadership Initiative in Sarcopenia (GLIS) [38] Another limitation stands in the multifactorial nature of sarcopenia. To obtain approval from regulatory agencies such as the FDA or EMA, a pharmacological treatment for sarcopenia should have the ultimate goal of reducing both mobility disability (or physical performance) and the rates of major health events. However, as reported by Rolland et al. [36], the current data from therapeutic trials highlight that the observed improvements in muscle mass and/or strength do not necessarily result in functional performance improvements. Approaches targeting either loss of muscle mass or strength may be insufficient to improve function, specifically due to the multifactorial nature of sarcopenia. Another limitation in clinical trials on sarcopenia is the endpoint selection. Primary outcomes vary a lot across studies. The definition of a core outcome set (COS) for sarcopenia, i.e. an agreed standardized set of outcomes that should be measured and reported, *as a minimum*, in all clinical trials in a disease [39], is still lacking. COS are encouraged by patient associations, scientific societies, and regulatory agencies for many reasons. Harmonizing outcomes by COS may ensure that outcomes selected in research are those that patients regard as the most important or relevant for them, may enhance transparency while achieving the highest methodological quality (e.g., avoiding selective outcome reporting and research-waste), and may streamline shared decision-making for trial and guideline developers, healthcare providers, scientific societies, funders, and regulatory agencies, focusing on prioritizing resources for patient-centered and scientifically robust interventions. Recently, Doza et al. [40]. published a systematic review pointing out the diversity of outcomes reported in clinical trials on sarcopenia. While the authors of this systematic review primarily aimed to identify clinical trials in sarcopenia using a PROM as primary or secondary outcomes, they nevertheless highlighted a huge heterogeneity of reported outcomes in the 17 randomized clinical trials (RCTs) identified. The nine different reported PROMs covered the assessment of various aspects, including quality of life, depressive symptoms, loneliness/social isolation, daytime sleepiness, insomnia impact, and sleep quality/disturbance. Only one sarcopenia-specific PROM, namely the SarQoL, was reported. The effect of sarcopenia-designed interventions on PROMs showed considerable heterogeneity, reinforcing the argument for the need for a COS for clinical sarcopenia trials.

The incorporation of PROMs into clinical trials is no longer in question, as Government regulatory agencies such as the FDA and EMA have advocated for their use in interventional studies [41,42]. As a reflection of this, the FDA has observed a 500% increase in the number of pre-market submissions that include PROMs between 2009 and 2015 [43]. Nevertheless, clarity regarding the principle of utilizing PROMs as co-primary or secondary endpoints is still lacking. For example, in the systematic review of Doza et al. [40], for example, which includes 17 RCTs, PROMs were mainly used as secondary outcomes. Nevertheless, five studies listed multiple primary outcomes among which one or more PROMs were listed. Finaly, only one RCT [44] used exclusively PROMs (i.e. depression, loneliness, and HRQoL) as primary endpoints. PROM measures are designed to be subjective and reflect patients' perspectives and experiences. PROMs do not replace other more objective measurements and are therefore expected to be used to complement clinical data. In this context, the European Society for Clinical and Economic Aspects of Osteoporosis, Osteoarthritis, and Musculoskeletal Diseases (ESCEO) working group recommended the use of co-primary endpoints, combining a measure of physical performance with PROMs in all Phase III clinical trials for sarcopenia. While using a PROM as the sole primary endpoint is not recommended, there is still room for debate regarding the use of PROMs as co-primary or secondary endpoints in clinical trials aimed at managing sarcopenia.

## 5 Final considerations

The ICSFR Task Force agreed and reaffirmed that using PROMs and PREMs as endpoints in clinical studies on sarcopenia may improve the understanding of a patient's status by providing information that may not be captured through biomedical methods due to the difficulty of observing certain aspects and their subjective nature. This approach may support healthcare professionals and future patients in choosing the most suitable treatment by giving a clearer view of personal experiences and identifying any unmet needs or areas in healthcare that require improvement.

However, the Task Force identified several research gaps and barriers that need to be addressed to expedite and improve the use of these outcome measures. In particular, comorbidities of sarcopenia with other conditions, including cognitive dysfunctions, depressive symptoms or metabolic syndrome, deserve greater attention. Indeed, sarcopenia has been associated with higher odds of cognitive impairment [45] with a higher risk of incident Alzheimer's disease dementia, mild cognitive impairment and cognitive decline [46]. Similarly, the prevalence of depression in patients with sarcopenia is higher than in the general population, and sarcopenia is associated with an increased risk of depressive symptoms [47,48]. Measuring PROMs (e.g., HRQoL) among people with cognitive deficits may be challenging as questionnaires need to be interviewer-administered, and proxies’ views and experiences are often necessary and may somehow affect the assessment. Accordingly, coexisting depressive and anxiety symptoms may sometimes influence how patients feel overall and the perceived quality of life. Based on these premises, disease-specific PROMs that have been successfully validated in the ideal settings of research protocols may be less sensitive to capture changes in experiences in patients living in the “real world”, where the presence of multiple comorbidities is the rule rather than the exception.

Along the same lines, PROMs should be culturally appropriate and valid to reliably capture the perceptions and experiences of individuals with different cultural backgrounds. Our societies and the populations of older community-dwelling individuals and older patients referred to healthcare services are increasingly multicultural. Nevertheless, culturally diverse individuals are still underrepresented in clinical trials, with relevant implications for the generalizability of the findings to practice. Moreover, they may have different experiences and face additional barriers along their journey in the healthcare system. In this regard, adopting translated and culturally validated PROMs may facilitate the inclusion of a wider range of participants, and adequately capture their perspectives and experiences. This may result in an increased representativeness of research populations and external validity of the findings.

## 6 Conclusion

The current approach to managing sarcopenia involves a multifaceted strategy to mitigate its impact on individuals’ health and well-being. Interventions are diverse, incorporating a blend of nutritional, exercise, and pharmacological strategies. Many of these approaches showed positive clinical benefits. However, the effective management of sarcopenia also requires a shift towards a more personalized and patient-centered approach. While PROMs implementation in the field of clinical trials in sarcopenia may remain challenging, the ICFSR task force believes that there are added value benefits in their use, namely by monitoring symptoms in individual patients, contributing to shared decision-making processes, supporting health economic decisions, and ultimately enhancing healthcare systems. Currently, three different PROMs specific to sarcopenia have been developed and are available for use by patients, clinicians, researchers, and pharmacological industries. Continued advancements in this area are crucial for improving patient outcomes and the overall effectiveness of sarcopenia management strategies.

## Conflict of interest

Charlotte Beaudart and Yves Rolland are stakeholders of SARQOL SRL, a spin-off of the University of Belgium, in charge of the interests of SarQoL, a specific health-related quality of life questionnaire for sarcopenia. However, they have never received any financial compensation for this role.

Roger A. Fielding is partially supported by the 10.13039/501100004477US Department of Agriculture (USDA), under agreement No. 58–8050–9–004, by NIH Boston Claude D. Pepper Center (OAIC; 1P30AG031679). Any opinions, findings, conclusions, or recommendations expressed in this publication are those of the authors and do not necessarily reflect the view of the USDA. RAF reports grant support from Lonza, Biophytis, National Institutes of Health, and USDA, scientific advisory board membership for Biophytis, Amazentis, Inside Tracker, Rejuventate Biomed, Aging in Motion, consultancies for Embion, Biophytis, Amazentis, Pfizer, Nestle, Rejuvenate Biomed.

David Cella is an uncompensated board member of the PROMIS Health Organization, a nonprofit organization dedicated to education about and advancement of PROMIS.

Johannes Grillari is co-founder and scientific advisor of Rockfish Bio AG, Vienna, Austria.

Other authors did not report any other conflicts of interest.
